# Identification and characterization of expressed retrotransposons in the genome of the *Paracoccidioides* species complex

**DOI:** 10.1186/s12864-015-1564-7

**Published:** 2015-05-12

**Authors:** Marco Aurélio Soares, Roberta Amália de Carvalho Araújo, Marjorie Mendes Marini, Luciana Márcia de Oliveira, Leonardo Gomes de Lima, Viviane de Souza Alves, Maria Sueli Soares Felipe, Marcelo Macedo Brigido, Celia Maria de Almeida Soares, Jose Franco da Silveira, Jeronimo Conceição Ruiz, Patrícia Silva Cisalpino

**Affiliations:** Departamento de Microbiologia, Instituto de Ciências Biológicas, Universidade Federal de Minas Gerais, 31270-901 Belo Horizonte, MG Brazil; Programa de Pós-graduação em Bioinformática, Instituto de Ciências Biológicas, Universidade Federal de Minas Gerais, 31270-901 Belo Horizonte, MG Brazil; Departamento de Microbiologia, Imunologia e Parasitologia, Escola Paulista de Medicina, Universidade Federal de São Paulo, 04023-062 São Paulo, SP Brazil; Laboratório de Biologia Molecular, Instituto de Ciências Biológicas, Universidade de Brasília, 70910-900 Brasília, DF Brazil; Laboratório de Biologia Molecular, Instituto de Ciências Biológicas, Universidade Federal de Goiás, 74001-970 Goiânia, GO Brazil; Grupo Informática de Biossistemas, Centro de Pesquisas René Rachou, FIOCRUZ-Minas, 30190-002 Belo Horizonte, MG Brazil; Departamento de Biologia Geral, Universidade Federal de Minas Gerais, Belo Horizonte, MG Brazil

## Abstract

**Background:**

Species from the *Paracoccidioides* complex are thermally dimorphic fungi and the causative agents of paracoccidioidomycosis, a deep fungal infection that is the most prevalent systemic mycosis in Latin America and represents the most important cause of death in immunocompetent individuals with systemic mycosis in Brazil. We previously described the identification of eight new families of DNA transposons in *Paracoccidioides* genomes. In this work, we aimed to identify potentially active retrotransposons in *Paracoccidioides* genomes.

**Results:**

We identified five different retrotransposon families (four LTR-like and one LINE-like element) in the genomes of three *Paracoccidioides* isolates. Retrotransposons were present in all of the genomes analyzed. *P. brasiliensis* and *P. lutzii* species harbored the same retrotransposon lineages but differed in their copy numbers. In the Pb01, Pb03 and Pb18 genomes, the number of LTR retrotransposons was higher than the number of LINE-like elements, and the LINE-like element RtPc5 was transcribed in *Paracoccidioides lutzii* (Pb01) but could not be detected in *P. brasiliensis* (Pb03 and Pb18) by semi-quantitative RT-PCR.

**Conclusion:**

Five new potentially active retrotransposons have been identified in the genomic assemblies of the *Paracoccidioides* species complex using a combined computational and experimental approach. The distribution across the two known species, *P. brasiliensis* and *P. lutzii,* and phylogenetics analysis indicate that these elements could have been acquired before speciation occurred. The presence of active retrotransposons in the genome may have implications regarding the evolution and genetic diversification of the *Paracoccidioides* genus.

**Electronic supplementary material:**

The online version of this article (doi:10.1186/s12864-015-1564-7) contains supplementary material, which is available to authorized users.

## Background

Transposable elements (TEs) have been found in virtually all eukaryotic species investigated to date [1,2] and may represent a significant portion of the genomes of living organisms. TEs can account for 80% or more of total genomic DNA in plants and comprise 45% and 20% of the genomes of metazoans and fungi, respectively [3,4]. TEs are DNA sequences with the ability to move from one genomic location to another and can be grouped in two classes according to whether their transposition intermediate is RNA (class I or retrotransposons) or DNA (class II or DNA transposons) [2,5].

Retrotransposons replicate by a “copy and paste” process, whereby the RNA intermediate is reverse-transcribed into double-stranded (ds) DNA by enzymes encoded by the TEs themselves. Elements belonging to class I are further divided into five orders based on their mechanistic features, organization and reverse transcriptase phylogeny: LTR retrotransposons, DIRS-like elements, Penelope-like elements, LINEs and SINEs [2,6]. LTR retrotransposons are the most widespread, especially those from the *Gypsy* and *Copia* superfamilies. Members of the LTR order usually encode two open reading frames (ORFs), one related to viral structural proteins (*gag)* and the second, known as *pol*, to a polyprotein composed of an aspartic protease (AP), a reverse transcriptase (RT), an RNase H (RH) and an integrase (IN) [2,6].

Although fungal genomes generally contain fewer repetitive sequences than higher eukaryotes, TEs are viewed as central agents in the evolution of fungal genomes [2,3,7]. In *Magnaporthe oryzae,* clusters of TEs were associated with increased rates of chromosomal rearrangements, gene duplication and gene evolution [8]. There is an apparent correlation between TE clustering and chromosomal polymorphism in *Fusarium oxysporum* [9], and in *Aspergillus niger*, recombination mediated by retrotransposons has led to inversions of genomic regions [10]. In *Verticillium dahliae,* an asexual plant pathogen, chromosomal rearrangements were found to be associated with retrotransposons, and the authors suggest that homologous recombination between highly similar copies of transposable elements might help to generate genetic diversity [11].

*Paracoccidioides* is a thermally dimorphic fungus that infects approximately 10 million people in Latin America, causing paracoccidioidomycosis, the most prevalent systemic fungal disease in this region and the deep mycosis responsible for the most deaths in immunocompetent individuals in Brazil [12,13]. Until 2006, the genus *Paracoccidioides* was believed to include only one species: *Paracoccidioides brasiliensis* [14]. It was only through Multilocus Sequence Typing (MLST) analysis that the genetic variability in this genus, formerly believed to be merely intraspecific and due to geographic polymorphism, was revealed. Four cryptic species, S1, PS2 and PS3, were identified from the *P. brasiliensis* complex [15,16], as well as the new species *Paracoccidioides lutzii* (originally called Pb01-like) [17,18].

Since 2009, it has been accepted that the *Paracoccidioides* genus is composed of four distinct phylogenetic lineages (S1,PS2, PS3 and Pb01-like), which vary in their virulence, culture adaptation and the different host immune responses they induce [15,19,20]. Strain Pb18 is a member of Species 1 (S1), which is composed of 38 isolates among the 65 studied and is distributed across Latin America [15]. The Pb03 isolate belongs to phylogenetic species 2 (PS2), which is composed of one Venezuelan and five Brazilian isolates among the 65 studied. The extensively studied clinical isolate Pb01 (*Paracoccidioides lutzii*) is phylogenetically distinct from the other strains [17].

A comparative genomic analysis of three *Paracoccidioides* species—two *P. brasiliensis* (Pb03 and Pb18) and one *P. lutzii* (Pb01)—identified all types of TEs. TEs correspond to approximately 8-9% of *P. brasiliensis* genomes and 16% of the *P. lutzii* genome [21]. We previously described a systematic survey of sequenced *Paracoccidioides* genomes for class II TEs (transposons) that resulted in the identification of eight families of DNA transposons. A detailed analysis of class II elements belonging to the Tc1/mariner superfamily revealed an unequal distribution among *Paracoccidioides* species and raised the possibility of the presence of active elements in the fungus genome [22]. TE insertions near genes can modify gene expression patterns, while insertions within genes can interrupt transcription and gene function [23]. In this work, we aimed to identify potentially active retrotransposons in the *Paracoccidioides* genome using a *Paracoccidioides* EST database to search for retrotransposons with evidence of transcription.

We expanded our search for class I TEs (retrotransposons) to identify and characterize potentially active retrotransposons in the *Paracoccidioides* species complex. We identified five different retrotransposons (four LTR-like and one LINE-like element) in the genomes of the three isolates. Whereas some DNA transposons are unequally distributed in the genomes of the *Paracoccidioides* species complex, retrotransposons are present in all of the genomes analyzed.

## Results

### Clustering, similarity and functional annotation of *Paracoccidioides* EST sequences

**Figure 1:**
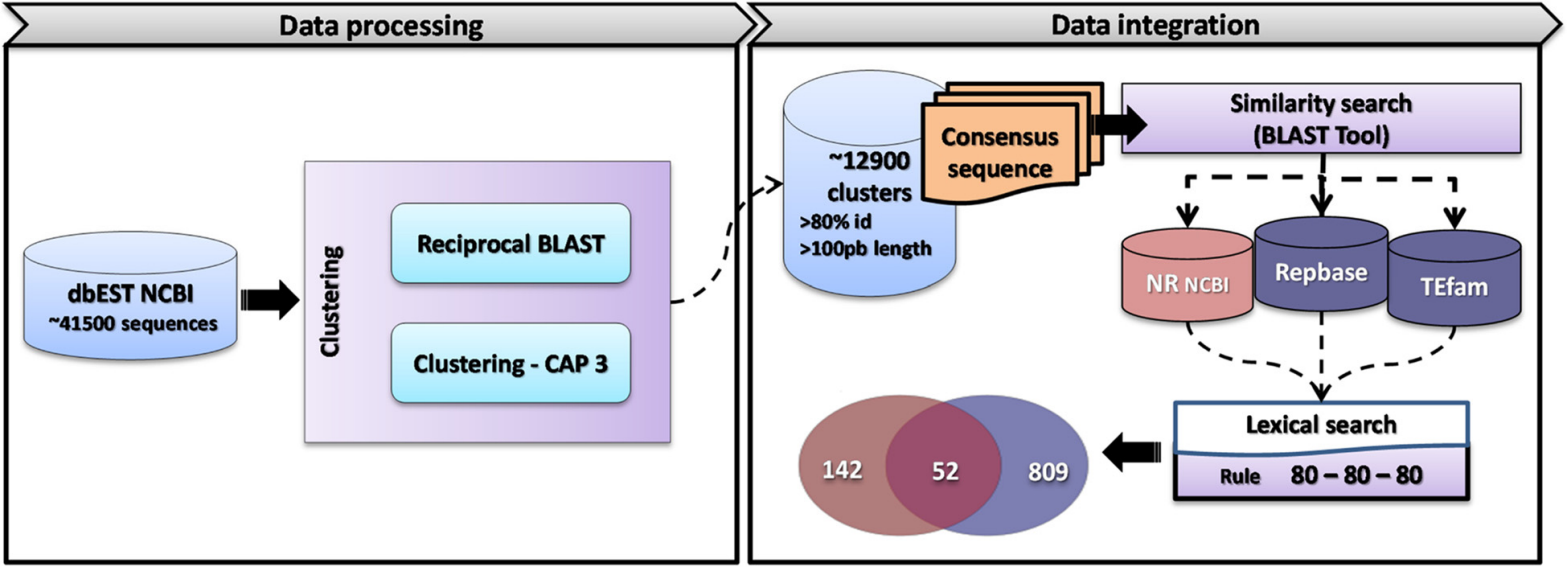
Consolidated workflow for the identification of Paracoccidioides EST sequences harboring retrotransposons. All EST sequences (41,558) available at dbEST (release 110111) for download from Paracoccidioides species were used to build a local database. The clustered approach adopted (reciprocal BLAST and CAP3) resulted in 12,922 clusters, which were used for further similarity searches against different databases (NR of proteins from NCBI, Repbase and TEfam). Blast hit descriptions were analyzed based on a lexical search approach (for details see Methods) and transposons matching rules suggested by Wicker and coworkers [2] for classifying eukaryotic TEs were adopted. A set of 52 EST sequences were found to be common between the results from similarity and lexical searches.

As shown in the workflow in Figure 1, the first step in the identification of active retrotransposons in the genomes of the *Paracoccidioides* species complex was to access the information contained in the EST database (http://www.ncbi.nlm.nih.gov/genbank/dbest/). A local database was built with 41,558 downloaded *Paracoccidioides* ESTs, which were then clustered, resulting in 12,922 sequence clusters distributed in 4,812 contigs and 8,110 singlets. The EST clusters were compared with sequences deposited in three databases: NR (the NCBI non-redundant protein database), TEfam and Repbase (Figure 1). Approximately 20% of the clusters (2,544/12,922) showed no similarity with any sequences in the NR database with the parameters set (see Methods section); Blast hit descriptions were analyzed based on a lexical search approach using specific keywords resulting in a first set of 142 EST sequences of putative retrotransposons (Figure 1). Further searches against specific databases identified 809 EST sequences common to the TEfam and Repbase databases (6.2% of all clusters) (Figure 1). A set of 52 EST sequences that were found to be common between the results from similarity and lexical searches were mapped in the *Paracoccidioides* genomes.

### Identification and characterization of *Paracoccidioides* retrotransposons

Employing the set of 52 EST clusters as queries against the *Paracoccidioides* genomes, five genomic sequences with characteristics of retrotransposons were identified and are referred to henceforth as RtPc (Retrotransposon *Paracoccidioides* complex) 1 to 5 (Table 1). All RtPc elements showed similarity with retrotransposons (Class I) (Table 1). Twenty-seven EST clusters were anchored to these five genomic sequences, each corresponding to a putative retrotransposon, distributed as follows: 18 clusters mapped to RtPc1, three to RtPc3 and RtPc5, two to RtPc4 and only one to RtPc2 (Additional file 1). The remaining 25 EST groups were mapped to genomic sequences with a large number of stop codons, preventing identification of complete copies of retrotransposons.

**Table 1:** **Classification of retrotransposons identified in** ***Paracoccidioides*** **genomes**

**Classification**				**Identity of the best hit**	
**Element**	**Class**	**Order**	**Superfamily/clade**	**Organism**	**% Similarity**
RtPc1	I (Retrotransposons)	LTR	*Gypsy*	*Aspergillus fumigatus*	64.9
RtPc2	I (Retrotransposons)	LTR	*Gypsy*	*Aspergillus nidulans*	64.4
RtPc3	I (Retrotransposons)	LTR	*Copia*	*Drosophila bipectinata*	68.6
RtPc4	I (Retrotransposons)	LTR	*Copia*	*Coccidioides posadasii*	62.6
RtPc5	I (Retrotransposons)	LINE	*I/ Tad-*like*	*Blumeria graminis*	63.9

*as mentioned in the Results, element LINE-*Tad*-like_RtPc5 was identical to the non-LTR retrotransposon of the *Tad-CgT* family, which was previously identified as element PbNLR1 by Novikova et al. [25].

**Figure 2:**
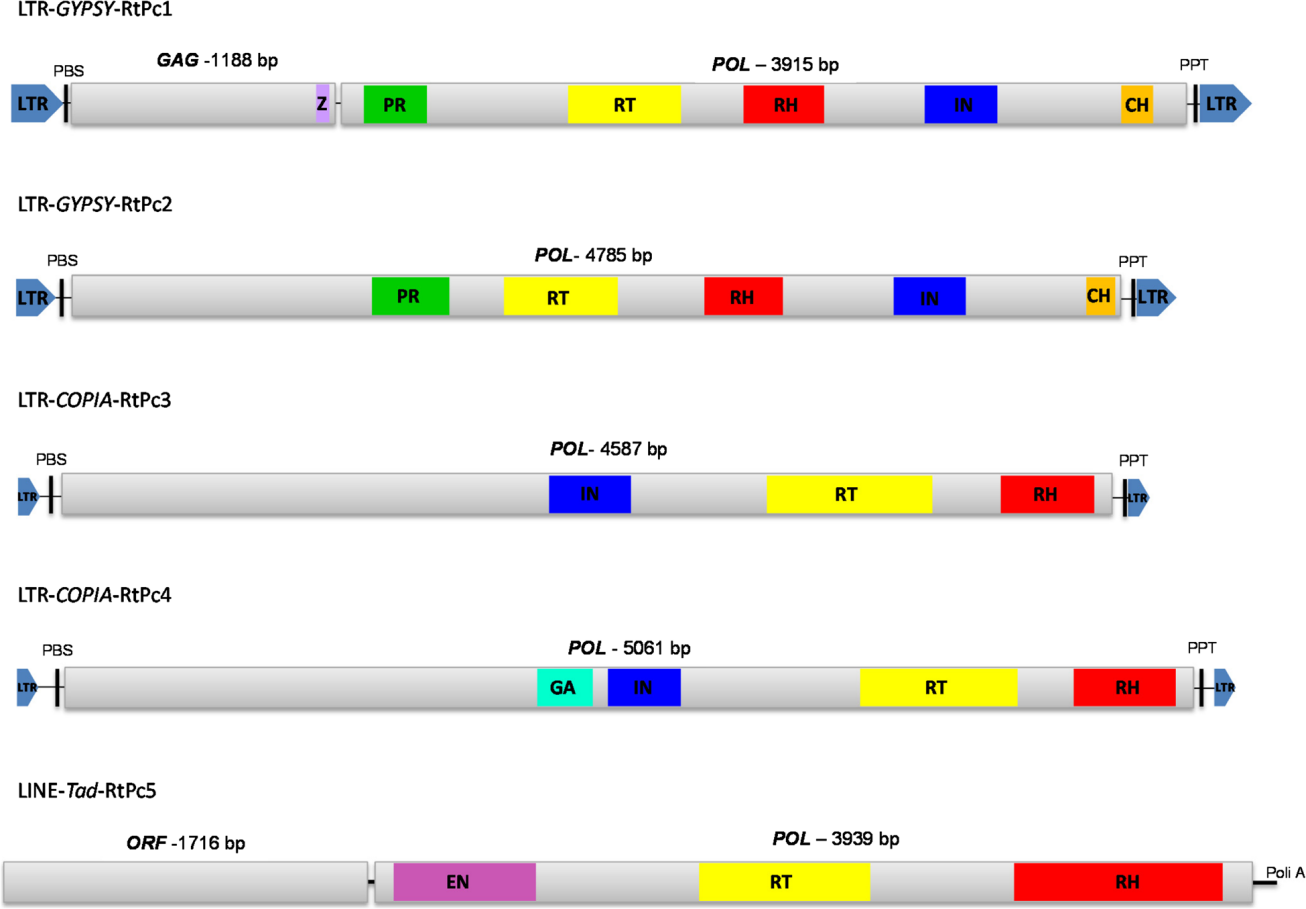
Structure and organization of RtPc elements in the *Paracoccidioides* complex. Schematic representations of complete RtPc elements are shown. The LTRs are represented by blue arrowheads and PBS/PPT by black bars. The domains are represented as follows: zinc finger – light purple; protease (PR) – green; reverse transcriptase (RT) – yellow; RNase H (RH) – red; integrase (IN) – blue; chromodomain (CH) – orange; and endonuclease (EN) – pink. The ORF with its respective size is represented above each element. Figures are not to scale.

Similarity searches were conducted to locate and perform the functional and structural annotation of intact, full copy elements in all supercontigs of *P. brasiliensis* (isolates Pb18 and Pb03) and *P. lutzii* (isolate Pb01), the three *Paracoccidioides* genomes (http://www.broadinstitute.org) [21]. Consensus sequences were generated by alignment of all intact copies of each retrotransposon (Additional file 2). We found that there is at least one intact copy of each RtPc element in one or more sequenced *Paracoccidioides* genome. The elements were classified (Table 1) based on the results of alignments with sequences of retrotransposons from the GIRI database (http://www.girinst.org/censor/index.php) using the criteria proposed by Wicker et al. [2] and Kapitonov et al. [24] (http://www.girinst.org/RTphylogeny/RTclass1/) [24]. We identified four RtPc elements belonging to the LTR order from the *Gypsy* (LTR-*Gypsy*-RtPc1 and LTR-*Gypsy*-RtPc2) and *Copia* (LTR-*Copia*-RtPc3 and LTR-*Copia*-RtPc4) superfamilies, and one element is a LINE retrotransposon (LINE-Tad-like-RtPc5) (Table 1, Figure 2) that we initially identified as a new type of fungal non-LTR retrotransposon related to the Tad clade.

### LTR retrotransposons of the superfamily *Gypsy* (LTR-*Gypsy*-RtPc)

The *Gypsy* elements identified in *Paracoccidioides* complex were 5412 to 5740 bp in length, with the coding regions flanked by LTRs. Primer binding sites PBS and PPT were also identified at the 5′ and 3′ ends of each element, respectively (Additional file 3, Figure 2). The element LTR-*Gypsy*-RtPc1 (5740 bp) has two ORFs corresponding to the gag gene, which is predicted to encode the proteins of the virus-like particle (VLP), and the pol gene, which encodes a polyprotein that gives rise to RNase H-reverse transcriptase, integrase and protease after processing. The Pol ORF was in a −1 frameshift in relation to the gag ORF. Coding sequences were flanked by 246-bp LTRs, with the nucleotides TG (initial) and CA (final). The first ORF (gag gene) encodes a 396-amino-acid (aa) protein with a conserved zinc finger domain (17 aa), and the second encodes a polyprotein that displays the following conserved domains: protease (99 aa), reverse transcriptase (176 aa), RNase H (126 aa), integrase (114 aa) and a chromodomain (52 aa). Full copies of LTR-*Gypsy*-RtPc2 (5412 bp) showed a single ORF that encodes a polyprotein with POL domains consisting of protease (96 aa), reverse transcriptase (178 aa), RNase H (123 aa) and integrase (113 aa). LTR-*Gypsy*-RtPc1 and LTR-*Gypsy*-RtPc2 shared 64.9% and 64.4% nucleotide sequence similarity, respectively, with retroelements identified in the fungus *Aspergillus nidulans* (Table 1).

### LTR retrotransposons from the *Copia* (LTR-*Copia*-RtPc) superfamily

The *Copia* elements identified in the *Paracoccidioides* complex were 5181 to 5685 bp long with the coding regions flanked by LTRs. The primer binding sites PBS and PPT were also identified at the 5′ and 3′ ends of each element, respectively (Additional file 3, Figure 2). Comparison with conserved domain databases showed that the element LTR-*Copia*-RtPc3 has a single ORF that encodes a 1528-aa protein with three conserved domains: integrase (126 aa), reverse transcriptase (253 aa) and RNase H (143 aa). The LTR-*Copia*-RtPc4 element had a single ORF with fused gag and pol sequences predicted to encode a 606-aa polyprotein with the following domains: Gag (87 aa), integrase (114 aa), reverse transcriptase (245 aa) and RNase H (160 aa). The elements LTR-*Copia*- RtPc3 and RtPc4 shared 68.6 and 62.6% nucleotide sequence similarity with retrotransposons identified in *Drosophila bipectinata* and in the fungus *Coccidioides posadasii*, respectively (Table 1).

### A Non-LTR retrotransposon similar to the *Tad* clade elements (LINE-*Tad*-RtPc5)

The complete element LINE-*Tad*-like-RtPc5 (5905 bp) contains two separate ORFs: the first encodes a protein (572 aa) with no similarity to any known protein, and the second, with a frameshift in relation to the first one, encodes a protein (1312 aa) that has conserved endonuclease (222 aa), reverse transcriptase (267 aa) and RNase H (145 aa) domains. This element has no LTRs, but a region corresponding to a poly A tail was identified (Additional file 3, Figure 2). LINE-*Tad*-RtPc5 shared 63.9% nucleotide sequence similarity with a retrotransposon identified in the fungus *Blumeria graminis* (Table 1). To assign this element to a specific clade, an automated tool (RtClass1, http://www.girinst.org/RTphylogeny/RTclass1/) [24] was employed that uses phylogenetic analysis of the RT domain protein. Based on the results from the RTclass1 tool, our LINE element clustered together with the *Tad1* clade, although the RtPc5 reverse transcriptase was indicated as belonging to an outgroup clade. In an attempt to find better information on non-LTR retrotransposons related to fungi, we uncovered an interesting report [25] in which the authors employed an *in silico* approach to survey the non-LTR retrotransposons in 57 fungal genomes, reporting more than 100 novel non-LTR retrotransposons and, importantly, describing two new clades, Inkcap and Deceiver. *P. brasiliensis* isolate Pb01 (now *P. lutzii*) was listed among the species searched by the authors, and three novel *Tad*-like elements, identified as PbNLR1, PbNLR2 and PbNLR3, were reported among the novel non-LTR elements harbored by this genome. Based on the phylogeny of the RT domains, the authors classified these novel *P. brasiliensis* non-LTR elements in the *Tad* clade under distinct families: *CgT*, *Ask1* and *Ask2*. On the basis of sequence identity, it was possible to establish the identity of the LINE-Tad-RtPc5 described here with the PbNLR1 element cited by [25], which is a *Tad-CgT* element.

### Distribution of retrotransposons in the *Paracoccidioides* species complex

After identifying at least one complete copy of each of the five elements, the RtPc sequences were used to identify intact and truncated copies in the sequenced *Paracoccidioides* genomes (Table 2). Overall, 538 copies of RtPc elements were found scattered throughout the genomes of the three isolates. The majority of RtPc elements (54.46%) were identified in *P. lutzii* (Pb01), and the remaining 245 copies were found in *P. brasiliensis* isolates (17.28% and 28.26% in Pb03 and Pb018, respectively) (Table 2). The distribution of copies of each element in the sequence supercontigs is shown in Additional file 4. Out of 538 retroelements identified in the *Paracoccidioides* genomes, 514 (95.54%) were truncated and only 24 (4.46%) were intact (Table 2). The distribution of truncated forms in *Paracoccidioides* species was as follows: 284 copies in *P. lutzii* (Pb01) and the remaining copies in *P. brasiliensis* isolates (141 copies in Pb18 and 89 in Pb03) (Table 2). Most of the intact elements were found in the Pb18 *P. brasiliensis* isolate (11/24) and Pb01 *P. lutzii* isolate (9/24), and four copies were found in the Pb03 *P. brasiliensis* isolate (Table 2). *Gypsy* elements were the most abundant retrotransposons in *Paracoccidioides* genomes, comprising approximately 53.34% (287/538) of the total retroelements identified in these species. Out of 111 copies of LTR-*Gypsy*-RtPc1, 57 were found in *P. lutzii*, followed by 39 and 15 in isolates Pb18 and Pb03, respectively. Four intact copies of RtPc1 were present in *P. lutzii*, and eight were present in Pb18 (Table 2). The LTR-Gypsy-RtPc2 element was the most abundant RtPc element (176/538) of all the retrotransposons identified in the *Paracoccidioides* genomes studied here. It is interesting to note that 56.81% of LTR-*Gypsy*-RtPc2 copies (100/172) were found in *P. lutzii* (isolate Pb01) and that all of these were truncated. Only one intact copy of this element was found in the genomes of *P. brasiliensis* isolates (Table 2).

**Table 2:** **Distribution of retrotransposons in** ***Paracoccidioides*** **species genomes**

	**Isolate Pb01**			**Isolate Pb03**			**Isolate Pb18**		
	**(** ***P. lutzii*** **)**			**(** ***P. brasiliensis*** **)**			**(** ***P. brasiliensis*** **)**		
**Elements**	**Copy**	**Intact**	**Truncated**	**Copy**	**Intact**	**Truncated**	**Copy**	**Intact**	**Truncated**
	**Number**			**Number**			**Number**		
LTR-*Gypsy*-RtPc1	57	4	53	15	0	15	39	8	31
LTR-*Gypsy*-RtPc2	100	0	100	6	0	6	70	1	69
LTR-*Copia*-RtPc3	5	0	5	10	3	7	3	1	2
LTR-*Copia*-RtPc4	66	0	66	28	0	28	9	1	8
LINE-*Tad-CgT*- RtPc5	65	5	60	34	1	33	31	0	31
Subtotal	293	9	284	93	4	89	152	11	141

*Copia* and LINE retrotransposons comprised 22.5% and 24.16% of all the retroelements in the *Paracoccidioides* genomes, respectively. Out of eighteen LTR-*Copia*-RtPc3 copies, ten were found in the isolate Pb03, followed by five in Pb01 and three in Pb18. As for the LTR-*Copia*-RtPc4 element, most copies were found in *P. lutzii* (n = 66), followed by *P. brasiliensis* isolates Pb03 (n = 28) and Pb18 (n = 9). For the LINE-*Tad*-RtPc5 element, again, most copies (n = 65) were identified in *P. lutzii (*Pb01), followed by isolates Pb03 (n = 34) and Pb18 (31). Most of these elements were truncated in the isolate Pb01 (92.3%) (Table 2).

### LTRs not associated with complete retrotransposons

In addition to intact and truncated elements, structural variations of LTR retrotransposons include solo LTRs, which together with LTR remnants are believed to be the result of unequal recombination and illegitimate recombination. We identified 468 copies of solo LTRs closely related *to Gypsy*- and *Copia*-RtPc elements; most of these were found in *P. lutzii* (n = 222), followed by *P. brasiliensis* isolates Pb18 (n = 164) and Pb03 (n = 81) (Additional file 4). Solo LTRs belonging to the *Gypsy* superfamily are far more abundant (2.6-fold) than those of *Copia*-like retrotransposons. The ratio of solo LTR sequences to intact elements was 13.4 in *Paracoccidioides* genomes, and the ratio of solo-*Gypsy* LTRs to intact elements was also higher than that for solo-*Copia* LTRs vs. intact elements (Additional file 4).

### The presence of RtPc elements in *Paracoccidioides* isolates of distinct phylogenetic origins

**Figure 3:**
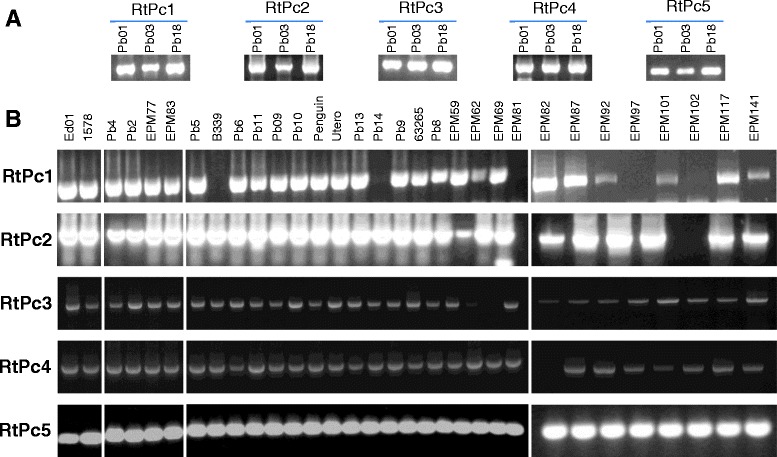
PCR screening for the reverse transcriptase domain of RtPc elements in phylogenetic lineages of *Paracoccidioides* species. Electropherogram results from PCR analysis of a phylogenetically diverse panel of *Paracoccidioides* species. PCR amplification of the reverse transcriptase domain of the five RtPc elements from the genomic DNA of 31 isolates of the three cryptic species of *P. brasiliensis* and three isolates of *P. lutzii.* Panel **A** shows the PCR amplification products for the five RtPc elements from the genomic DNA of the three isolates sequenced in the Broad Institute FGI (Pb01, P03 and Pb18). Panel **B** shows the amplification products for the 5 RtPc elements from the genomic DNA of 31 *Paracoccidioides* isolates. The first block is composed by two P. lutzii isolates. The second block by four P. brasiliensis, the first two being PS2 and the latter two PS3 isolates. In the third block the first 13 isolates are P. brasiliensis S1 and the latter four are *Paracoccidioides* spp. The fourth block, at right, is composed by eight *Paracoccidioides* spp. Oligonucleotide primer pairs are given in Additional file 6.

To investigate the occurrence of RtPc elements, a segment of the coding sequence for reverse transcriptase was PCR amplified from the genomic DNA of 31 isolates, including Pb01-like *P. lutzii* isolates and isolates belonging to the *Paracoccidioides* phylogenetic lineages S1, PS2 and PS3 from the *P. brasiliensis* complex (Table 3, Figure 3). Reverse transcriptase was present in 24 isolates. The identity of the amplicons was confirmed by sequencing a 300-bp fragment corresponding to the coding region for the reverse transcriptase of each of the five elements (isolates Pb01, Pb03 and Pb18) (data not shown). The element LINE-Tad-RtPc5 was present in all isolates. No Gypsy element was found in isolates EPM81 and EPM102. No correlation was found between the distribution patterns of RtPc elements and the *phylogeny* of *Paracoccidioides* lineages.

**Table 3:** ***Paracoccidioides*** **isolates used in this study**

**Isolate**	**Origin**	**Country**	**Phylogenetic species** ^**c**^
Pb01^af^	clinical	Brazil – Goiás	*P. lutzii*
Ed01^ef^	clinical	Brazil – Goiás	*P. lutzii*
1578^ef^	clinical	Brazil – Goiás	*P. lutzii*
Pb03^adeg^	chronic PCM^b^	Brazil – São Paulo	*P. brasiliensis -* PS2
Pb4^deg^	chronic PCM	Brazil – São Paulo	*P. brasiliensis -* PS2
Pb2^deg^	chronic PCM	Venezuela	*P. brasiliensis -* PS2
EPM77^e^	clinical	Colombia	*P. brasiliensis -* PS3
EPM 83^e^	chronic PCM	Colombia	*P. brasiliensis -* PS3
Pb18^ade^	chronic PCM	Brazil – São Paulo	*P. brasiliensis -* S1
Pb5^g^	chronic PCM	Brazil – Paraná	*P. brasiliensis -* S1
B339^deg^	chronic PCM	Brazil – São Paulo	*P. brasiliensis -* S1
Pb6^deg^	chronic PCM	Brazil – Paraná	*P. brasiliensis -* S1
Pb11^deg^	acute PCM	Brazil – Paraná	*P. brasiliensis -* S1
Pb09^d^	clinical	Venezuela	*P. brasiliensis -* S1
Pb10^deg^	acute PCM	Peru	*P. brasiliensis -* S1
Penguin^de^	penguin feces	Uruguay	*P. brasiliensis -* S1
Utero^f^	chronic PCM	Argentina	*P. brasiliensis -* S1
Pb13^deg^	acute PCM	Brazil – Goiás	*P. brasiliensis -* S1
Pb14^deg^	acute PCM	Brazil – São Paulo	*P. brasiliensis -* S1
Pb9^deg^	chronic PCM	Brazil – São Paulo	*P. brasiliensis -* S1
63265^e^	acute PCM	Argentina	*P. brasiliensis -* S1
Pb8^d^	chronic PCM	Brazil – São Paulo	*P. brasiliensis -* S1
EPM59	chronic PCM	Venezuela – Caracas	ND
EPM62	acute PCM	Venezuela – Caracas	ND
EPM69	chronic PCM	Venezuela – Caracas	ND
EPM81	clinical	Colombia – Medellin	ND
EPM82	chronic PCM	Colombia – Bogotá	ND
EPM87	acute PCM	Argentina – Entre Rios	ND
EPM92	Clinical	Brazil – Piauí	ND
EPM97	chronic PCM	Brazil – Maringá	ND
EPM101	Armadillo	Brazil –Ibiá	ND
EPM102	Armadillo	Brazil – Ibiá	ND
EPM117	Armadillo	-	ND
EPM141	clinical	Brazil – Paraná	ND

^**a**^ *P. brasiliensis* isolates used in the genome sequencing project – Broad Institute of MIT and Harvard.

^**b**^PCM – paracoccidioidomycosis.

^**c**^Phylogenetic Species grouped in accordance with Matute et al. [15]^**d**^, Teixeira et al. [17]^**e**^ and Marini et al. [22]^**f**^.

^**g**^Isolate was also used by Morais et al. [59].

### Genomic organization and transcription of RtPc elements

**Figure 4:**
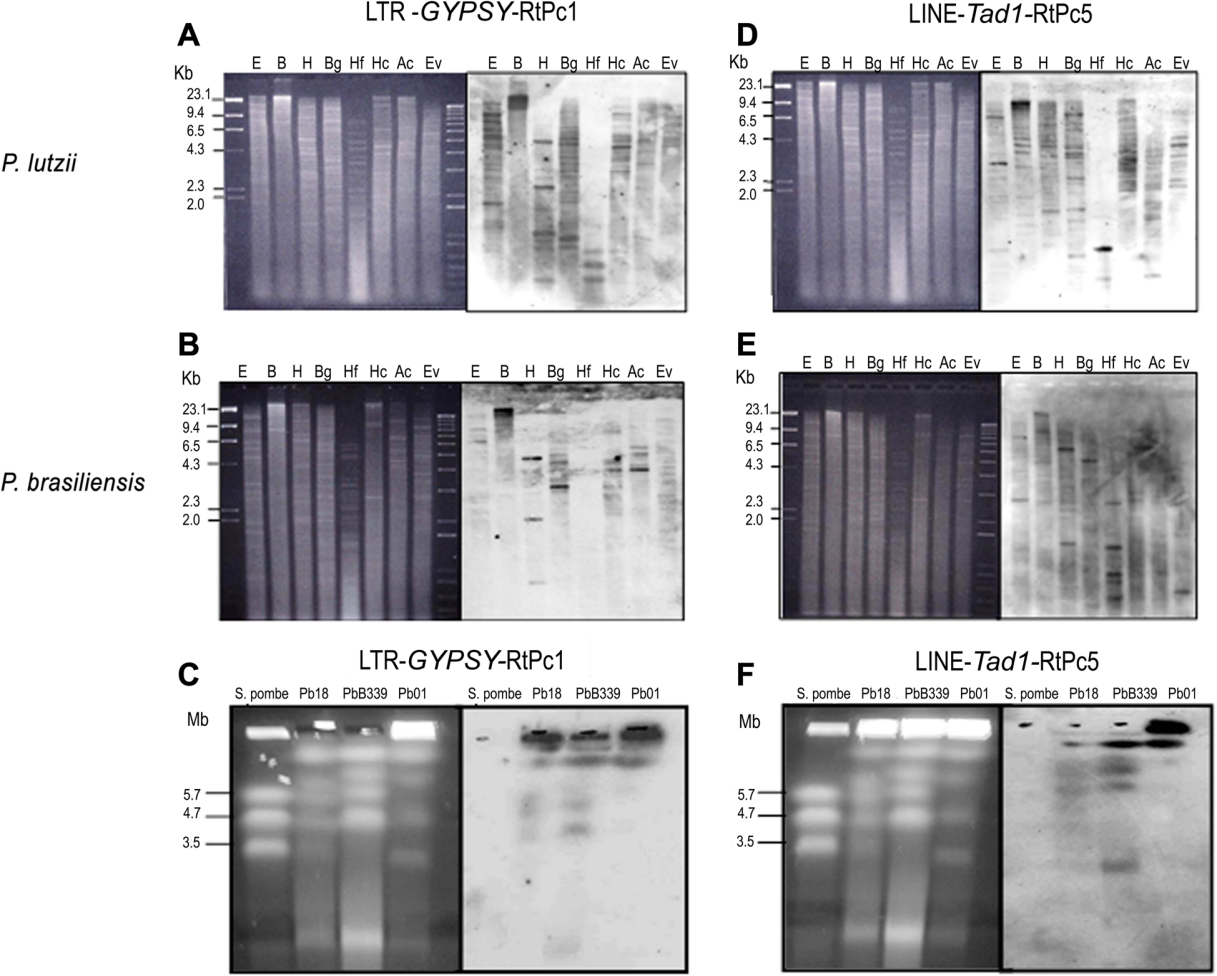
Genomic Southern blot analysis and chromosomal distribution of RtPc1 and RtPc5 elements. Genomic DNA from *P. lutzii* **(panels A and D)** and the Pb18 *P. brasiliensis* isolate **(panels B and E)** were digested with restriction enzymes, blotted onto nylon membranes and hybridized with RtPc1 **(panels A** **and B)** and RtPc5 **(panels D and E)** probes derived from the transcriptase reverse region of each element. The restriction enzymes used were EcoRI **(E)**, BamHI **(B)**, HindIII **(H)**, BglII(Bg), HinfI (Hf), HincII (Hc), AccI (Ac) and EcoRV (Ev). Chromosomal distribution of RtPc1 **(panel C)** and RtPc5 **(panel F)** elements in *P. lutzii* and *P. brasiliensis*. The molecular karyotypes of *P. lutzii* and *P. brasiliensis* (B339 and Pb18) are shown on the left. Chromosomal bands were separated by PFGE and stained with EtBr. The autoradiograms from Southern hybridizations using the RtPc1 and RtPc5 probes derived from the transcriptase reverse region are shown on the right.

We also analyzed the genomic organization of RtPc elements by Southern blot hybridization using probes corresponding to the retrotransposons LTR *Gypsy*-RtPc1 and LINE-*Tad*-RtPc5. Figure 4 shows the results obtained using genomic DNA from *P. lutzii* (Pb01) and *P. brasiliensis* (Pb18). As expected from the *in silico* analysis, the LTR-*Gypsy*-RtPc1 probe hybridized to multiple genomic fragments from *P. lutzii* (Pb01) and *P. brasiliensis* (Pb18), confirming the polymorphic nature and abundance of this element. The number and signal intensity of hybridizing fragments identified in Pb01 was higher than in Pb18, confirming the variation in the copy number of LTR-*Gypsy*-RtPc1. The hybridization patterns obtained with the element LINE-*Tad*-RtPc5 indicate that these elements were more abundant in the *P. lutzii* genome.

LTR-*Gypsy*-RtPc1 and LINE-*Tad*-RtPc5 elements were mapped to the chromosomal bands of isolates Pb01 and Pb18 (Figure 4, panels C and F), which had been separated by PFGE. Pb01 and Pb18 showed distinct karyotype profiles with four and five chromosomal bands, respectively. Isolate B339 was used as a reference for chromosomal band size. The LTR-*Gypsy*-RtPc1 probe hybridized to three and four chromosomal bands in isolates Pb01 and Pb18, respectively (Figure 4, panel C). The LINE-*Tad*-RtPc5 probe hybridized to three and two chromosomal bands in isolates Pb18 and Pb01, respectively (Figure 4, panel F).

**Figure 5:**
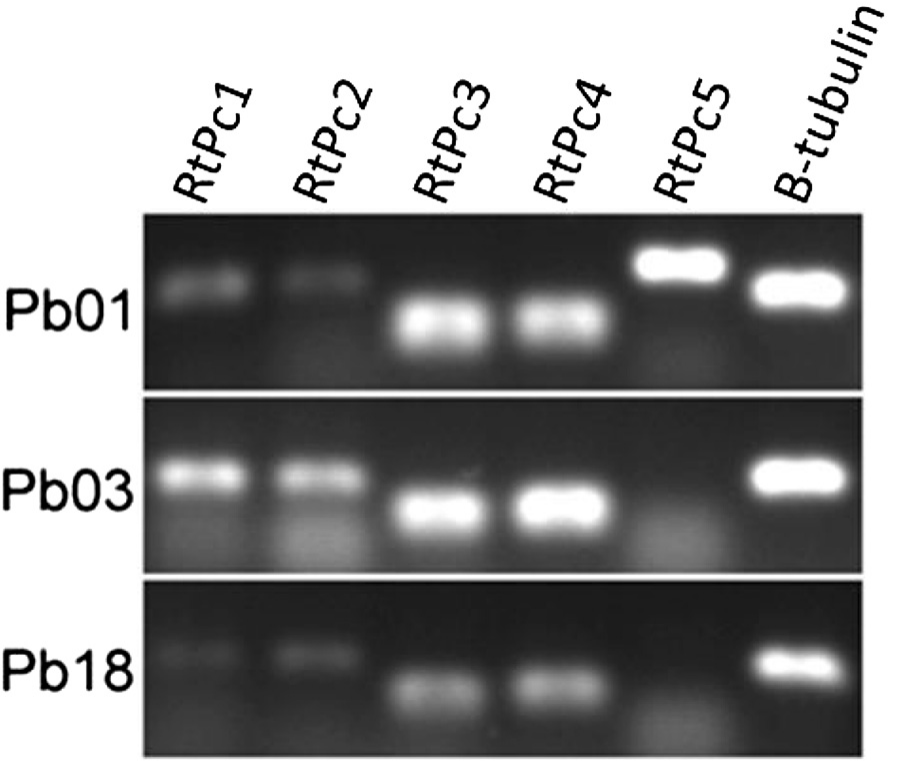
RtPc transcript analysis. Total RNA from Pb01, Pb03 and Pb18 was used to detect mRNAs corresponding to the reverse transcriptase region of RtPc elements. Reverse transcription data was normalized to β-tubulin.

To detect transcripts of RtPc elements, semi-quantitative RT-PCR was performed using cDNAs from isolates Pb01 (*P. lutzii*), Pb03 and Pb18 (*P. brasiliensis*, PS2 and S1). Figure 5 shows that the five retrotransposons were transcribed in the yeast form of *P. lutzii* (Pb01), but only RtPc1, RtPc2, RtPc3 and RtPc4 were transcribed in the yeast form of *P. brasiliensis* (Pb03 and Pb18).

### Phylogenetic analysis of RtPc elements

**Figure 6:**
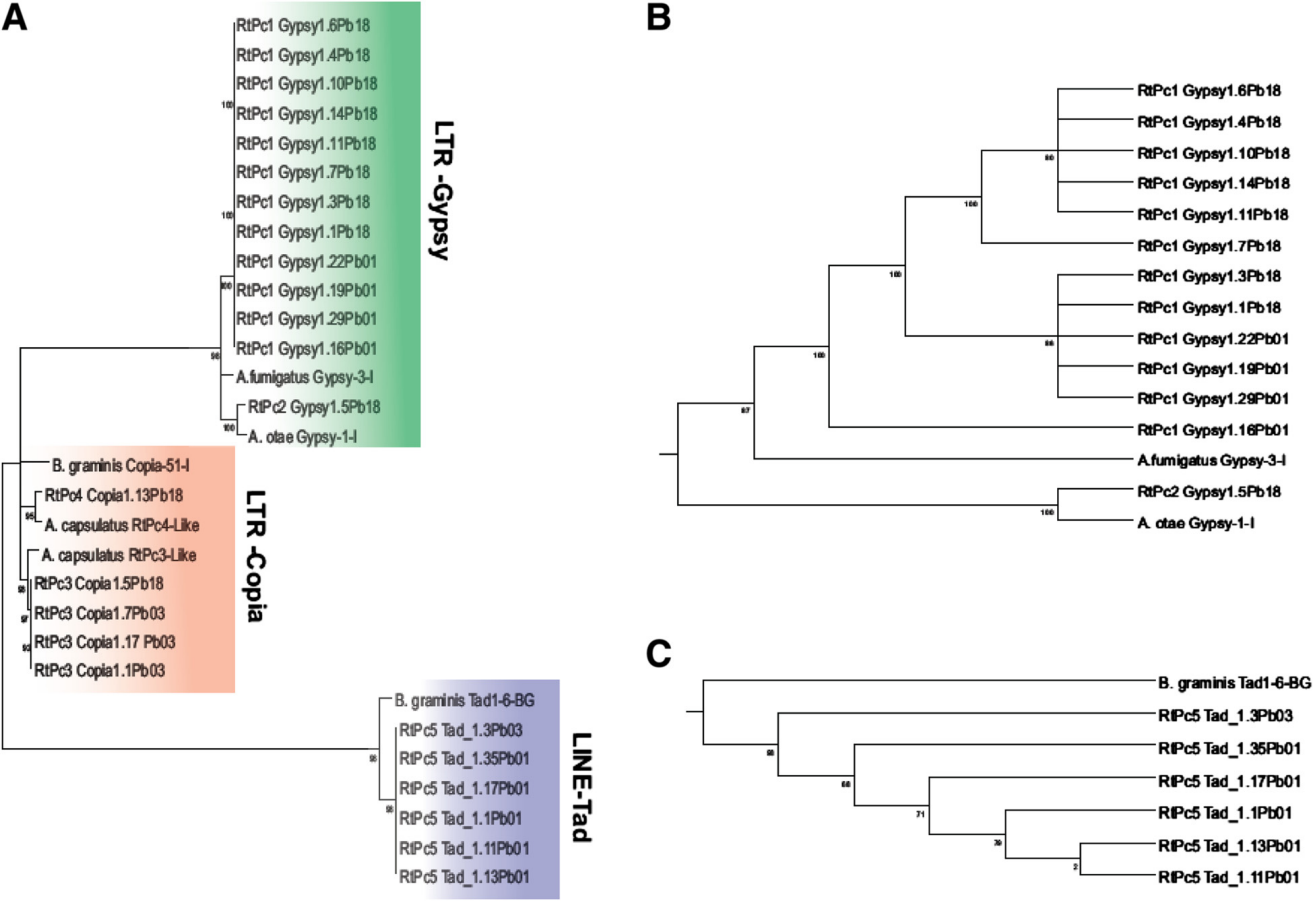
Phylogenetic analysis of RtPc elements and related fungal retrotransposons. Phylogenetic tree of all intact RtPc elements using nucleotide sequences of the reverse transcriptase domain of *P. brasiliensis* (Pb 03 e Pb18) and *P. lutzii* (Pb01) with Bayesian inference. Trees were rooted with the other subfamilies. LTR elements from *Aspergillus fumigatus* (Gypsy-3-I_AF), *Arthroderma otea* (Gypsy-1-I_Ao), *Ajellomyces capsulatus*(AAJI01001759.1) *Blumeria graminis* (Copia-11_BG-I),*Ajellomyces capsulatus* (XM_001540167.1) and *Blumeria graminis* (Tad1-16-BG) were used as outgroups (see Method). **A** shows the phylogenetic tree of all 24 RtPc intact elements identified in Pb01, Pb03 and Pb18. LTR-*Gypsy*-RtPc1 and LINE-*Tad*-RtPc5 trees are shown with more detail in **B** and **C**, respectively.

The reverse transcriptase domains from the 24 complete elements were employed to establish the phylogenetic relationship between RtPc elements derived from each of the two species of the genus *Paracoccidioides*, *P. brasiliensis* (isolates Pb18 and Pb03) and *P. lutzii* (isolate Pb01). Intact copies of LTR-*Gypsy*-RtPc1 and LINE-*Tad*-RtPc5 have been identified in *P. brasiliensis* and *P. lutzii*. In the phylogenetic tree (Figure 6A), three major clusters can be distinguished. One group contains the LTR-*Gypsy* elements, the second contains LTR-*Copia* elements and the third contains the LINE elements, all strongly supported by high posterior probabilities, thus confirming the classification of these elements. The phylogenetic tree for the LTR-*Gypsy*-RtPc1 element (Figure 6B) showed 3 clusters, two of which were composed of species-specific sequences from *P. brasiliensis* (Pb18) and *P. lutzii* (Pb01). The first branch consisted of sequences exclusively from *P. brasiliensis* (Pb18), comprising 6 of the 8 intact copies found in this species (supercontigs 1.4, 1.6, 1.7, 1.10, 1.11 and 1.14); the central branch was composed of two RtPc sequences from *P. brasiliensis* (Pb18, supercontigs 1.1 and 1.3) and three from *P. lutzii* (Pb01, supercontigs 1:22, 1:19, 1:29 and 1:16), and a high similarity was observed among the five elements inside this cluster (Figure 6B). The grouping of elements from these two different species illustrates the degree of similarity between these elements and suggests a common ancestry. The third branch harbored only one *P. lutzii* sequence (supercontig 1.16) (Figure 6B). This pattern suggests that the RtPc1 elements from *P. brasiliensis* and *P. lutzii* share sequences that were present in a relatively recent common ancestor, thus supporting the hypothesis of pre-speciation emergence of RtPc1 insertions. The RtPc3-Copia elements clustered in very close branches and showed a high sequence similarity, which could be explained by the low number of copies analyzed and by the fact that intact copies were only identified in *P. brasiliensis* lineages Pb03 and Pb18. However, for the LINE-*Tad*-RtPc5 element (Figure 6C), it was possible to observe a species-specific grouping, although only a single copy of RtPc5 has been analyzed in *P. brasiliensis* (isolate Pb03). Thus, despite the LINE-*Tad*-RtPc5 elements from P*. brasiliensis* and *P. lutzii* being located in separate branches, they nevertheless are phylogenetically close (Figure 6C).

Based on the assumption that the two LTRs accumulate point mutations independently, the insertion time of an LTR retrotransposon could be estimated based on variations or sequence differences between the two LTRs ends, where would have been identical at the time of insertion [26]. Very low nucleotide divergence was observed in the LTRs of the 18 intact RtPc elements (Additional file 5). Considering that the analyzed elements are intact and most likely active, the estimated divergence between LTRs suggested recent insertion events in the genomes of *P. brasiliensis* and *P. lutzii.*

## Discussion

Our group previously described a systematic survey of class II TEs (transposons) in the sequenced *Paracoccidioides* genomes that identified eight families of DNA transposons [22]. Here, we expand our search for class I TEs (retrotransposons) to identify and characterize potentially active retrotransposons in the *Paracoccidioides* species complex. To achieve this goal, a combined computational and experimental approach was applied to map these elements in the sequenced *Paracoccidioides* genomes (*P. lutzii* – Pb01 and *P. brasiliensis* – Pb03 and Pb18). We identified five different retrotransposon families (four LTR-like and one LINE-like element) in the genomes of the three isolates. Although some DNA transposons are unequally distributed in the three *Paracoccidioides* genomes, with some lineage-specific elements, retrotransposons are spread over all of the genomes analyzed. However, our data reveal several interesting characteristics of retrotransposon organization in *Paracoccidioides* species. For instance, *P. brasiliensis* and *P. lutzii* species harbor the same retrotransposon lineages but differ from one another in the number of copies present. *P. lutzii* contains 1.9 to 3.1 times more retrotransposons than *P. brasiliensis* isolates Pb18 and Pb03, respectively. Differences in LTR content were also observed between closely related species, such as those in the *Coccidioides* genus [27]. Another remarkable characteristic was the high number of truncated elements. For example, all *Gypsy*-RtPc2 and *Copia*-like insertions were degenerated in *P. lutzii.* Despite the differences in number and integrity between the five retrotransposon families, they were most likely acquired before the speciation event that gave rise to *P. brasiliensis* and *P. lutzii*, in contrast to some DNA transposons that appear to have been acquired after this event [22].

Desjardins et al. [21] performed a comparative genomic analysis and reported a high number of TEs in *Paracoccidioides* genomes. Mobile elements account for 16% of the *P. lutzii* genome and 7.7% and 9.2% of the genomes of *P. brasiliensis* isolates Pb03 and Pb18, respectively. A total of 971 retrotransposons were identified in the *P. lutzii* genome, a two-fold expansion compared with the *P. brasiliensis* genomes (338 and 460 elements in Pb03 and Pb18, respectively). In our work, we identified a smaller number of retrotransposons than previously described [21], with a total of 293 in Pb01, 93 in Pb03 and 152 in Pb18. This outcome may be explained by the fact that we searched for potentially active elements in an EST database, and given that highly defective elements are not represented in EST databases, they may not have been identified with this strategy.

LTR retrotransposons are classified into five superfamilies, of which *Copia* and *Gypsy* are found in fungal genomes, with *Gypsy* being the most abundant [2,3,27]. As expected for this type of genome, *P. lutzii* contains more *Gypsy* than *Copia* elements, and in isolate Pb18, there were nine times more of the former than of the latter. In isolate Pb03, however, we identified 38 *Copia* and 21 *Gypsy* elements. In the global analysis of transposable elements performed by Desjardins et al. [21], slightly more *Gypsy* elements (85 copies) than *Copia* elements (80 copies) were found. In the three genomes they studied, there were more LTR retrotransposons than LINE-like elements.

Active transposable elements can be highly mutagenic, inserting into coding regions and leading to chromosomal breaks and rearrangements. Genomes tend to resist the expansion of TEs, and many organisms have developed genome defense systems that repress the activity of mobile elements [28-30]. Several mechanisms could be responsible for retrotransposon removal in *Paracoccidioides* genomes. One such mechanism could involve the erosion of LTR retrotransposons through the accumulation of point mutations or minor deletions. This accumulation could be due to repeat-induced point mutations (RIPs), as described in *Neurospora* [31], in which retrotransposons were inactivated by the accumulation of point mutations generated by an increased number of stop codons resulting from G-C to A-T transitions [27]. The presence of multiple stop codons was observed in the majority of retrotransposons identified in *Paracoccidioides* genomes in our study; in fact, only 5% of the identified elements corresponded to intact copies. Analysis of GC content indicated an especially high AT content in LTR-*Gypsy*-RtPc2 and LTR-*Copia*-RtPc4 retrotransposons, which have the highest proportion of truncated elements. Desjardins et al. [21] also reported a lower CG content (37% – 39%) in transposon sequences than in the rest of the *Paracoccidioides* genome. The generation of solo-LTRs as a by-product of recombination between flanking LTRs of the same element could also be responsible for the deterioration of these elements. This seems to be the main mechanism driving the deterioration of Ty3/*Gypsy* elements in *Anopheles gambiae* [32]. A large number of solo LTRs were found in all of the genomes analyzed in our study, indicating that a similar mechanism may have occurred in *Paracoccidioides* and could have played a role in the generation of the genomic plasticity observed in these fungi.

We used PCR to analyze the distribution of RtPc elements in 34 *Paracoccidioides* isolates of different geographic origin, four of which were environmental. Only LINE-*Tad*-RtPc5 was identified in all isolates, the elements LTR-*Gypsy*-RtPc2, LTR-*Copia*-RtPc3 and LTR-*Copia*-RtPc4 were absent in only one isolate, and the element LTR-*Gypsy*-RtPc1 could not be amplified in five isolates. The accumulation of mutations in several elements most likely explains why we failed to amplify these elements by PCR in some isolates. We could not establish any correlation between the presence of the elements and the origin of the isolate. However, the wide distribution of transposons in most *Paracoccidioides* isolates indicates that they may have been present in the common ancestor before the speciation event.

TEs can be inserted into various regions of the eukaryotic genome, including the flanking regions of euchromatic genes. There are several cases of TE insertions acting in genes regulatory pathways, which can interfere with their activation or inactivation [4,33-35], suffering higher selective pressures. Hence, the high similarity observed between all RtPc 1, RtPc3 and RtPc5 elements suggests that the elements described in the present work are still active or under purifying selection, otherwise several mutations would be expected. Through phylogenetic analysis, we can infer that the two copies of the element LTR-*Gypsy*-RtPc1 present in the genome of *P. brasiliensis* (isolate Pb18) group together with elements of the genome of *P. lutzii* (isolate Pb01). The retrotransposition mechanism of LTR, “copy and paste”, is equivalent to gene duplication; hence, the emergence of specific RtPC1 lineages in Pb01 and Pb18 isolates could occur concomitantly with the RtPc1 copies present in the common ancestor. If this process occurred independently in *P. brasiliensis* and *P. lutzii*, we should see a distribution similar to that shown in the phylogenetic trees obtained in the present work (Figure 6). Fungal genomes exhibit a low rate of evolution [36], which could explain why even the specific lineages are still very similar to the non-specific elements. Then, the emergence of *P. brasiliensis* RtPc1-specific lineages due to the accumulation of mutations can occur concurrently with the maintenance of copies of RtPc1 similar to those present in the common ancestor of *P. lutzii* and *P. brasiliensis* [37]. However, for the LINE element RtPc5 it was possible to observe a species-specific grouping, although only a single copy of RtPc5 from *P. brasiliensis* (isolate Pb03) has been analyzed. Therefore, we suggest that the RtPc elements were present in the ancestral lineage shared by *P. lutzii* (Pb01) and *P. brasiliensis* (Pb03 and Pb18) and that the inclusion of these elements must have occurred at this point, especially for LTR-*Gypsy-* RtPc1 and LINE-*Tad*-RtPc5. The abundant number and high degree of similarity of these elements indicate that they have undergone a recent amplification process within the genomes of these species. This observation is consistent with the life-cycle of a transposable element [38].

TEs may have two main effects on the host genome: insertion into a new chromosomal locus when the element transposes and ectopic recombination. Two copies of the same element in different chromosomal locations may act as sites for homologous recombination, the effects of which can range from small inversions to major chromosomal rearrangements, such as duplications, deletions or translocations [39,40]. A significant percentage of the *Paracoccidioides* genome consists of transposable elements that could serve as sites for homologous recombination. In organisms without sexual reproduction, homologous recombination between TEs might be associated with the generation of genetic diversity [11]. Recent evidence suggests that *Paracoccidioides* species have the potential to undergo sexual reproduction, although to date a sexual reproduction stage has not been described [41,42]. We identified potentially active retrotransposons in *Paracoccidioides* genomes and also described potentially active DNA transposons. Recently, Shankar and coworkers [43] reported a high expression of retrotransposons in *P. lutzii* Pb01 during the dimorphic transition and were able to suppress the transition when the fungi were treated with 17β-estradiol. *Paracoccidioides* dimorphism can be triggered by 37°C temperatures in hosts or in culture, and the morphological differentiation that takes place during the mycelial-to-yeast transition is considered to be vital to the pathogenesis of PCM [43,44]. The transposition of active elements can have major mutational effects. TE insertion near or within a gene may alter or destroy the activity of the gene in several ways, ranging from total inactivation to changes in expression levels or alternative splicing [7]. A recently conducted study in *Schizosaccharomyces pombe* showed that the transcription of the LTR retrotransposon Tf1 was induced by heat treatment, and genes that were induced by heat could be activated by Tf1 integration. The authors speculate that Tf1 integration has the potential to improve the survival of individual cells exposed to environmental stress [45].

## Conclusions

A combined computational and experimental approach was effective in identifying and characterizing five new potentially active retrotransposons in the genomic assemblies of the *Paracoccidioides* species complex. All elements have been found in both *P. lutzii* and *P. brasiliensis* (S1, PS2 and PS3). This finding suggests that the RtPc elements would have already been present in a common ancestor. Full-length, potentially functional autonomous elements were characterized, and transcripts of all five elements have been detected. Considering the growing body of evidence in the literature indicating the major importance of TEs, it would be interesting to investigate the impact of active retrotransposons on the biology of these dimorphic pathogenic fungi.

## Methods

### Bioinformatics and data analysis

*Paracoccidioides brasiliensis* expressed sequence tags (ESTs) were retrieved from the NCBI dbEST database (http://www.ncbi.nlm.nih.gov/genbank/dbest/ NCBI EST database release 110111) (Figure 1). The search resulted in 41,558 EST sequences that were stored in a local database. The clustering process involved an initial step employing a reciprocal blast (all versus all blastn) of EST sequences followed by an assembly using the CAP3 program [46]. ESTs were assembled into clusters of overlapping sequences at least 100 bp with 80% identity. The resulting contigs and singlets, hereafter referred to as clusters, were used to identify regions with similarity to retrotransposons. The blastx and tblastx algorithms [47] were used to compare these groups with sequences from three sequence databases: a) NR, the NCBI non-redundant protein database; b) Repbase, the GIRI (Genetic information Research Institute) database of repetitive elements (http://www.girinst.org/repbase/index.html). The analyses were performed using default parameter values and an E-value of 10^−6^. All of the results collected after the various analyses performed in the characterization of each transposon family were stored in a hyperlinked Excel spreadsheet. Each line of the spreadsheet resents a cluster containing a variable number of sequences containing the follow descriptions: query id; seq size; annotation term; e-value and raw score; % identity; and % match length (Data not shown). A specific lexical search using eight keywords (retrotransposon, retroviruses, reverse transcriptase, gag, endonuclease, RNase H, integrase, Copia-like) was conducted. To get a start on clusters for the queries against *Paracoccidioides* genomes, the EST clusters common to both the NR and specific databases were identified (Figure 1).

### Databases

The following databases and versions were used: a) the *P. brasiliensis* genomic sequences (isolates Pb01, Pb03 and Pb18) from the *Paracoccidioides brasiliensis Sequencing project, Broad Institute of MIT and Harvard* (release 12–2010 (http://www.broadinstitute.org/annotation/genome/paracoccidioides_brasiliensis/Downloads.html); b) the NR protein database from NCBI (release 10.2011-186); c) TEfam (release VB2010-12); and d) Repbase (release RepBase16.11).

### Annotation and classification of sequences

Structural and functional annotations were performed using Artemis: Genome Browser and Annotation Tool (http://www.sanger.ac.uk/Software/Artemis/) and PERL scripts developed to analyze and parse the results. Retrotransposons were manually annotated based on the similarities of the sequences to proteins stored in the SeqDBLite fraction of the Gene Ontology database (http://www.geneontology.org), Pfam (Protein Families - http://pfam.sanger.ac.uk/), CDD (Conserved Domain Database www.ncbi.nlm.nhi.gov/Structure/cdd/wrpsb.cgi) and files used by InterProScan, as well as based on the presence of specific signatures, such as long terminal repeats - LTRs. The LTR regions, which are specific features of retrotransposons, were identified by the Tandem Repeat Finder (TRF) [48] and LTR_FINDER programs [49]. Functional annotation was confirmed by manual inspection. Classification of retrotransposons was based on nomenclature previously established for classifying TEs [2,24]. Retrotransposons were classified by class, subclass, order and superfamily. In this report, two retrotransposons were considered to belong to the same family if they shared 80% (or more) sequence similarity in at least 80% of the aligned sequences in either their coding region or internal domain or within their long terminal repeats in segments of at least 80 bp (80-80-80 rule) [2]. The RTclass1 computational tool was used to detail the classification of non-LTR retrotransposon elements (http://www.girinst.org/RTphylogeny/RTclass1/) [24]. All consensus sequences for each putative element were submitted to Repbase and compared using the Repeat Masking algorithm to identify related elements (http://www.girinst.org/censor/index.php).

### Phylogenetics analysis and evolution of RtPc elements

Regarding the phylogenetic inference, to standardize and avoid different substitution rates among the sequences, only the Reverse Transcriptase (RT) domains from complete elements were considered. Individual alignments were created for each RtPc family sequence using Muscle 4.04 [50], and the results were manually inspected. Each consensus sequence was used in sequence similarity searches using BLAST [47] against nr/nt NCBI databases to identify the closest related fungal retrotransposon. Additionally, Repbase was used to identify putative orthologous sequences. The best evolutionary model was predicted by jModelTest [51], and the Bayesian inference (BI) was evaluated with BEAST 2.1.3 [52]. During the BI analysis process, the following parameters were adopted: a) GTR + I + Γ model (a four-category gamma distribution) and b) analyses were performed for 100,000,000 generations with a sampling frequency of 100 and with a burnin of 2,500,000. Divergence time dates were used as priors when present in uTimetree.org [53] with the following parameters: a) a Calibrated Yule tree structure and b) a lognormal strict clock with nucleotide substitution rate 6.3 × 10–3 bp/Myr [36]. *Aspergillus fumigatus* (Repbase:*Gypsy*-3-I_AF), *Arthroderma otea* (Repbase:Gypsy-1-I_Ao), *Ajellomyces capsulatus* (Genbank:AAJI01001759.1) *Blumeria graminis* (Repbase:Copia-11_BG-I), *Ajellomyces capsulatus* (Genbank:XM_001540167.1) and *Blumeria graminis* (Repbase:Tad1-16-BG) sequences were used as outgroups for RtPc 1, 2, 3, 4 and 5, respectively.

### *P. brasiliensis* isolates and growth conditions

The *Paracoccidioides* isolates are listed in Table 3. Fungal isolates were maintained by periodic subculturing using YPD medium (5 g.L^−1^ yeast extract, 10 g.L^−1^ bactopeptone, 15 g.L^−1^ dextrose and 15 g.L^−1^ agar, pH 6.3) at 35°C – 37°C [54]. To obtain cells in the exponential phase [55], yeast cells were subcultured three times in YPD medium at 5-d intervals. The entire growth of two culture slants was inoculated into Erlenmeyer flasks containing 50 mL of YPD broth, placed on a reciprocating shaker at 120 rpm and grown for 5 – 7 d at 35°C. The cells were washed with sterile Milli-Q water and frozen in liquid nitrogen prior to DNA extraction.

### DNA/RNA extraction

Total DNA was extracted from the yeast culture following previously described protocols involving maceration of frozen cells [56] with minor modifications. Total RNA was extracted from the yeasts cultures as previously reported [57].

### Genomic DNA PCR analysis

PCR reactions were performed on 10 ng of DNA in a 20-μL reaction mixture containing 1.5 mM MgCl_2_ (50 mM), 100 mM of each dNTP, 100 pmol of each oligonucleotide and 1 unit of Taq DNA Polymerase (Promega). Each PCR was performed in a thermocycler (Eppendorf) for 35 cycles. The cycling conditions were as follows: denaturing at 94°C for 1 min, annealing at the Tm for each primer for 30 s, and extension at 72°C for 1 min. At the end of the 35^th^ cycle, the heat-denaturing step was omitted and extension was allowed to proceed at 72°C for 5 min. The primers corresponding to each retrotransposon are listed in Additional file 6.

### Cloning and sequencing

Fragments of approximately 300 bp in length related to the reverse transcriptase of each retrotransposon were amplified by PCR, and the amplicons were cloned into the pGEM-T Easy vector (Promega) and transformed into *Escherichia coli* DH5 competent cells. The nucleotide sequences of the DNA clones were determined by the dideoxynucleotide chain termination method, using the BigDye 3.1 Kit (Applied Biosystems).

### Blotting analysis

For Southern blot analysis, DNA samples digested with different restriction enzymes (*Eco*R I, *Bam*H I, *Hind* III, *Bgl* II, *Hinf* I, *Hinc* II, *Acc* I, *Nco* I, *Sal* I and *Eco*R V) were separated by electrophoresis on 0.8% agarose gels and stained with ethidium bromide (0.5 μg/mL). The agarose gels were incubated with 0.25 M HCl for 30 min, denatured with 0.5 M NaOH/1.5 M NaCl for 30 min, neutralized with 1 M Tris-base/0.5 M NaCl for 30 min and transferred onto nylon membranes in 20 × SSC (0.15 M NaCl/ 0.015 M sodium citrate) for three hours. The membranes were prehybridized (1% BSA, 500 mM NaH_2_PO_4_, 1 mM EDTA and 7% SDS) [58] for 2 h at 48°C and then hybridized overnight at the same temperature with a ^32^P-labeled probe. The probes used in the hybridization experiments were derived from the internal region of the reverse transcriptase segment of each element. Following hybridization, the membranes were washed four times in 2 × SSC containing 0.1% SDS at room temperature before being exposed to X-ray film.

For chromoblot analysis, the separation of chromosome-sized *P. brasiliensis* DNA molecules by PFGE (pulsed field gel electrophoresis) was performed as described by Feitosa et al. [54]. The agarose gels were incubated with 0.25 M HCl for 30 min, denatured with 0.5 M NaOH/1 M NaCl for 30 min, neutralized with 1 M Tris-base/0.5 M NaCl for 20 min and transferred onto nylon membranes in 20 × SSC (0.15 M NaCl/0.015 M sodium citrate). The membranes were prehybridized in a solution containing 50% formamide, 5 × SSC, 0.5% Denhardt’s solution, 0.1 mg/mL salmon sperm DNA and 0.1 mg/mL tRNA at 42°C for 1 h and then hybridized overnight at the same temperature with a ^32^P-labeled probe. The probes used in the hybridization experiments were derived from the internal region of each element, including the ORF. Following hybridization, the membranes were washed three times (30 min each) in 2 × SSC containing 0.1% SDS at 42°C, 1 × SSC containing 0.1% SDS and 0.1 × SSC containing 0.1% SDS at 56°C before being exposed to X-ray film.

### Transcriptional analysis (RT-PCR)

For RT-PCR, reverse transcription reactions were performed with 2 μg of total RNA. cDNA strands were prepared using oligo-dT primers and the standard protocol of Improm II reverse transcriptase (Promega). After cDNA synthesis, PCR reactions were performed with 1 μL of cDNA in a 20-μL reaction mixture containing 1.5 mM MgCl_2_ (50 mM), 100 mM of each dNTP, 100 pmol of oligonucleotides from the reverse transcriptase region and 1 unit of Taq *Platinum* DNA Polymerase (Invitrogen). Amplification was performed for 30 cycles. Cycling conditions were as follows: denaturing at 94°C for 1 min, annealing at 50°C for 30 s and extension at 72°C for 1 min. β-tubulin was used as an internal control.

## Additional files

Additional file 1:
**Structure and organization of RtPc elements of the** ***Paracoccidioides*** **complex.** Schematic representations of RtPc complete elements are shown. The LTRs are represented by orange arrows and PBS/PPT by black arrows. The domains are represented as follows: zinc finger – gray; protease – green; reverse transcriptase – yellow; RNase H – red; integrase – light blue; chromodomain – dark-blue; and endonuclease – pink. The ORF with its respective size is represented above each element. Arrows below the schematic representation indicate EST groups anchored to the element. The figures are not to scale. Additional file 2:
**Consensus sequences of RtPc elements. Consensus nucleotide sequences of the RtPc elements identified.**
Additional file 3:
**Characteristics of RtPc elements identified in** ***Paracoccidioides*** **genomes.**
Additional file 4:
**List of all RtPc insertions in** ***Paracoccidioides*** **genomes.** The complete list of all RtPc insertions, indicating the position of each element in the genomes of *Paracoccidioides lutzii* (Pb01) and *Paracoccidioides brasiliensis* (Pb03 and Pb18). Additional file 5:
**Estimation of the insertion time of LTR retrotransposon elements in the** ***Paracoccidioides lutzii*** **and** ***Paracoccidioides brasiliensis*** **genomes.**
Additional file 6:
**Primer sequences used to amplify RtPc elements by PCR and** **RT-PCR.** Table with the sequences of the primers used to amplify RtPc elements by PCR and RT-PCR.
