# Supplementary material for: Identification and characterization of expressed retrotransposons in the genome of the Paracoccidioides species complex
Source: BMC Genomics. 2015 May 12;16(1):376. doi: 10.1186/s12864-015-1564-7 (PMC4427930; doi:10.1186/s12864-015-1564-7)
Supplement: Additional file 1: — Structure and organization of RtPc elements of the Paracoccidioides complex. Schematic representations of RtPc complete elements are shown. The LTRs are represented by orange arrows and PBS/PPT by black arrows. The domains are represented as follows: zinc finger – gray; protease – green; reverse transcriptase – yellow; RNase H – red; integrase – light blue; chromodomain – dark-blue; and endonuclease – pink. The ORF with its respective size is represented above each element. Arrows below the schematic representation indicate EST groups anchored to the element. The figures are not to scale. [file 12864_2015_1564_MOESM1_ESM.docx]

|  | | **RT-PCR** | | **PCR** | | **Probe** | |
| --- | --- | --- | --- | --- | --- | --- | --- |
| **Primer** | | Sequence 5'-3' | Melting Temperature | Sequence 5'-3' | Melting Temperature | Sequence 5'-3' | Melting Temperature |
|  |  |  |  |  |  |  |  |
| **LTR-Gypsy-RtPc1** | F | CAGGTCCTCAGACTCAGGGTC | 61,0 °C | GTGAGAGGTTTGAGATGAA | 49,0 °C | ACATTCGGCAGGGGTTCCATC | 59,9 °C |
|  | R | CCTACGCCTCCCCAATCCTG | 65,0 °C | CTTTGCTAGGTTGGCTTC | 51,0 °C | GGGGCCTGCCATCTCACAAT | 60,3 °C |
| **LTR-Gypsy-RtPc2** | F | GCGAATTGGACAGGCTAAATGG | 65,3 °C | GCAAAAGCTGATGTGATTCC | 52,3 °C | TCAGCGAGACTCTACAGCG | 58,0 °C |
|  | R | CCGAGCAGAAGTCGTCCAGATAT | 64,0 °C | GCTTGCGAGAGAAGAAGGCA | 57,7 °C | CCCGTAGACGCATAAGCA | 55,5 °C |
| **LTR-Copia-RtPc3** | F | TACGGGTCGTATGCCGCCATA | 65,0 °C | CTAGGAGGGATGCCGCAC | 58,0 °C | TCTCTAACTTGCTCGCTAACG | 54,0 °C |
|  | R | CTCCACGGACATTGGTTTGTGC | 65,3 °C | TACTTGGTTGAGGATGGCGAA | 56,3 °C | TGTGATGGCGTATCTTCATGG | 55,0 °C |
| **LTR-Copia-RtPc4** | F | CCCAGATATGGCGCTTCTACG | 62,0 °C | AAGAACCTGCCTTCCGCCGC | 63,3 °C | TTAACCGCCTCAAGCAGATTG | 55,5 °C |
|  | R | CCGATACAACTGGTGCGAATG | 64,0 °C | TGCAGAGAGCCAACCCCCGA | 63,8 °C | GCATCAATTCGCATACCAGAT | 53,0 °C |
| **LINE-Tad1-RtPc5** | F | TCAGACGAAGGATGCTGCGG | 64,0 °C | TCAGACGAAGGATGCTGCGG | 64,0 °C | ACCACACGGGCCTTTGAGAACTAC | 61,1 °C |
|  | R | CCTGGAGAGTAACAACATTCCC | 61,0 °C | CCTGGAGAGTAACAACATTCCC | 61,0 °C | AAGACGGGGGCAAGACAGAGGAGA | 63,6 °C |
| **Β-tubulina** | F | TCGTCCGTCGTGAGGCTG | 65,0 °C |  |  |  |  |
|  | R | CAGAAGGTCTCATCAGCGTGCTC | 61,0 °C |  |  |  |  |
| **L35** | F | AATCGCTGGAGGTGCCG | 63,0 °C |  |  |  |  |
|  | R | CACAGCATATTTCCGCTGAGG | 63,0 °C |  |  |  |  |
