# Supplementary material for: Identification and characterization of expressed retrotransposons in the genome of the Paracoccidioides species complex
Source: BMC Genomics. 2015 May 12;16(1):376. doi: 10.1186/s12864-015-1564-7 (PMC4427930; doi:10.1186/s12864-015-1564-7)
Supplement: Additional file 2: — Consensus sequences of RtPc elements. Consensus nucleotide sequences of the RtPc elements identified. [file 12864_2015_1564_MOESM2_ESM.pptx]

## Slide 1
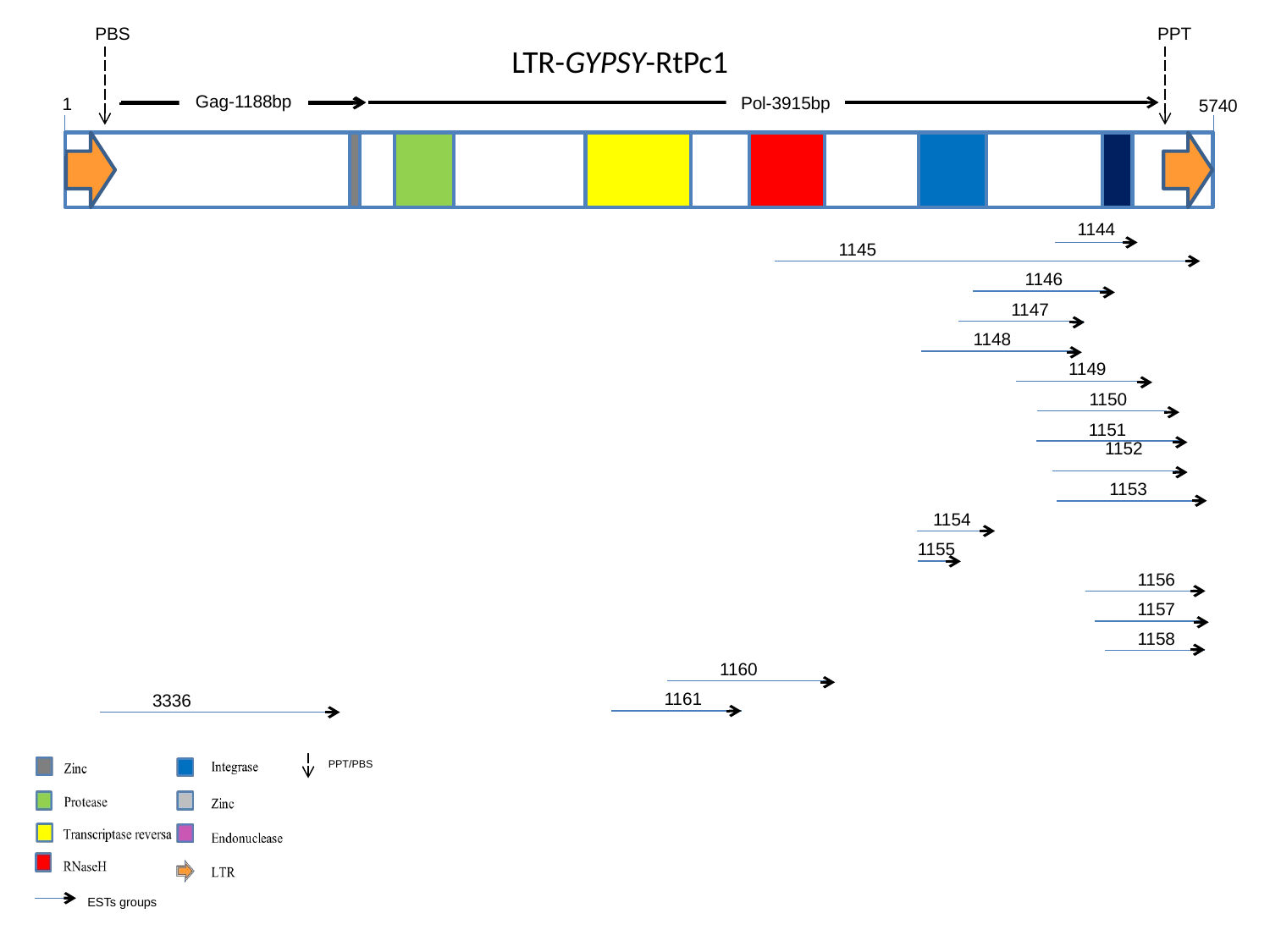

PBS
PPT
LTR-GYPSY-RtPc1
Gag-1188bp
Pol-3915bp
1
5740
1144
1145
1146
1147
1148
1149
1150
1151
1152
1153
1154
1155
1156
1157
1158
1160
1161
3336
PPT/PBS
ESTs groups

## Slide 2
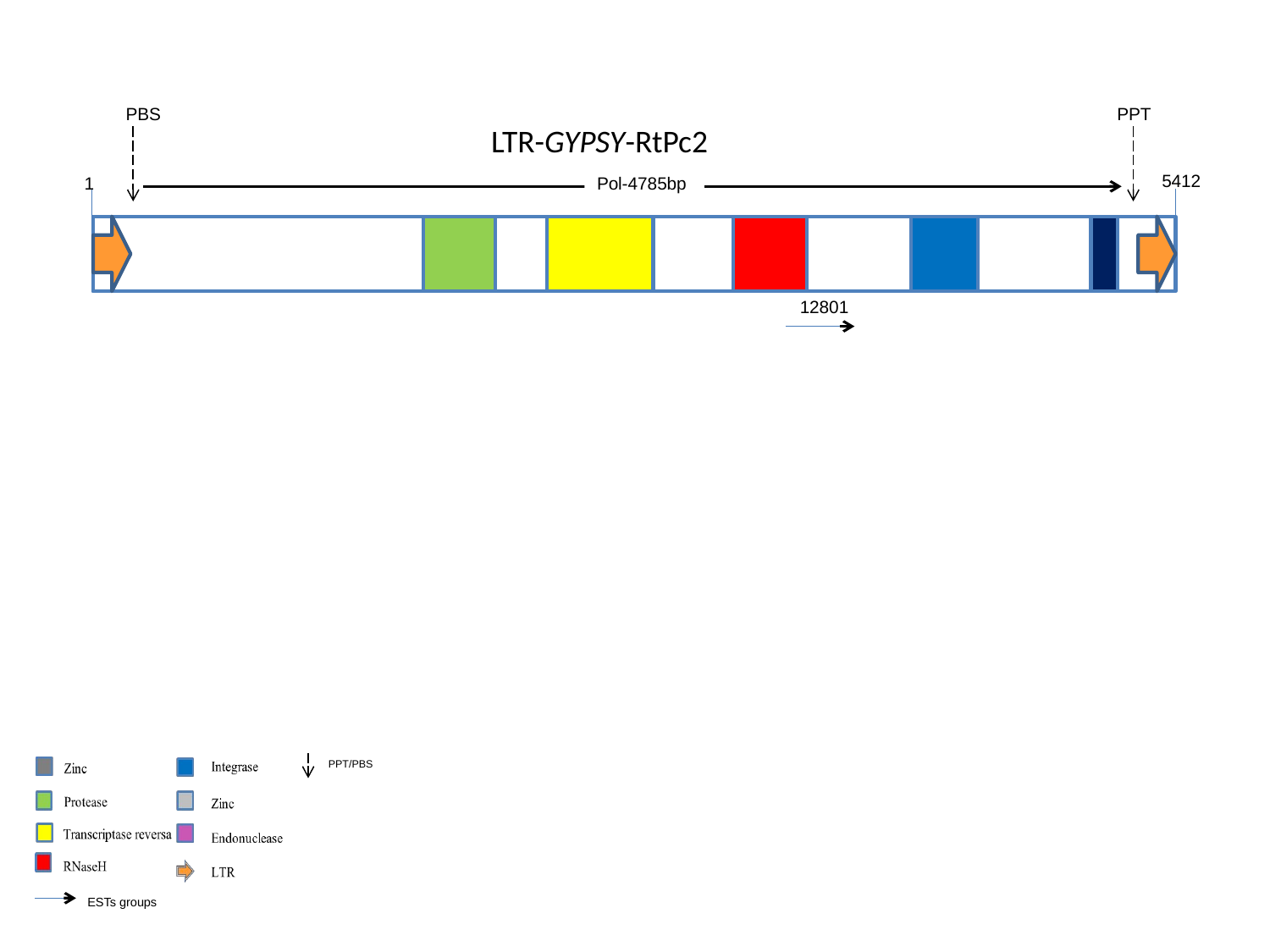

PBS
PPT
LTR-GYPSY-RtPc2
5412
1
Pol-4785bp
12801
PPT/PBS
ESTs groups

## Slide 3
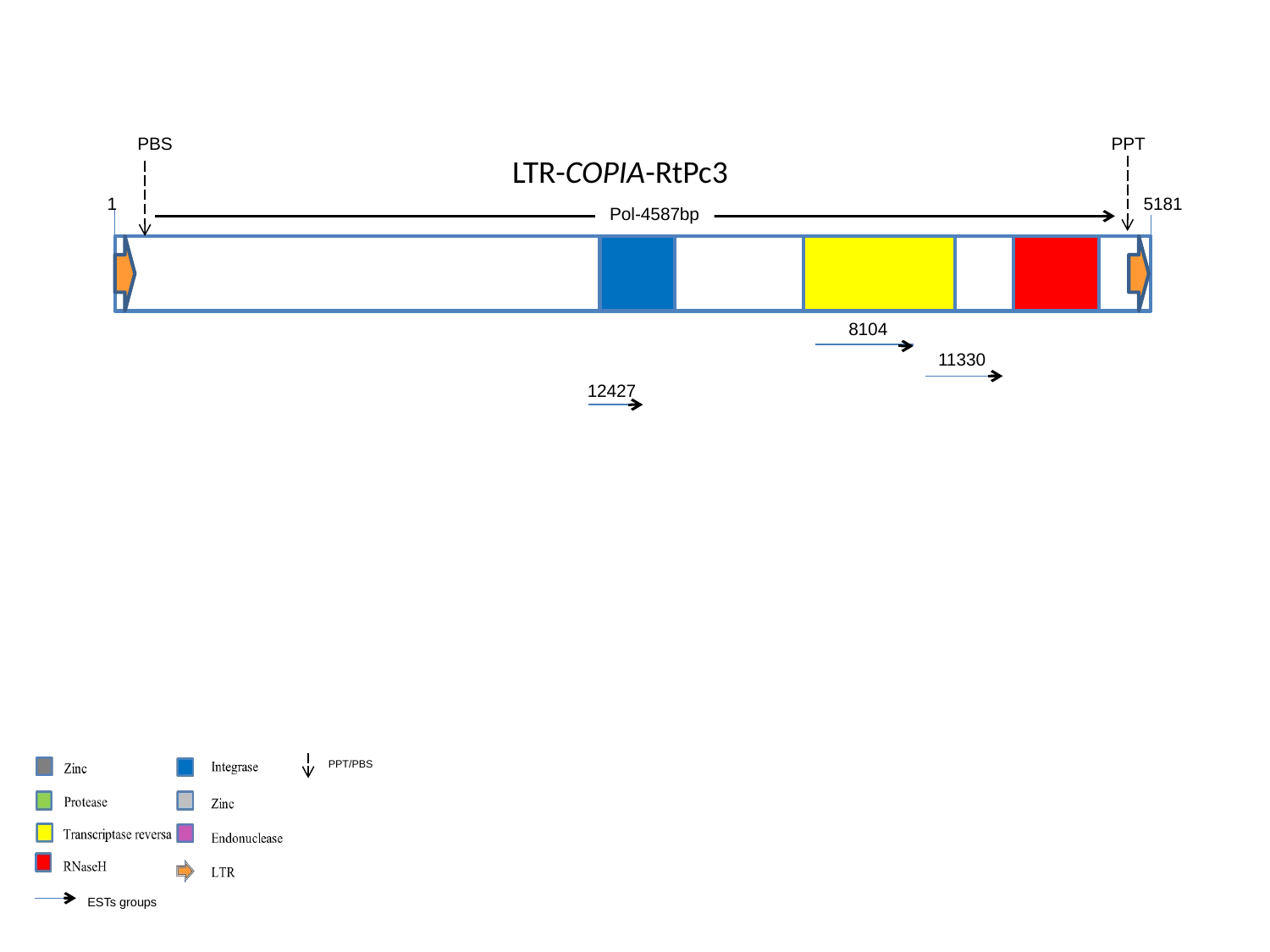

PBS
PPT
LTR-COPIA-RtPc3
1
5181
Pol-4587bp
8104
11330
12427
PPT/PBS
ESTs groups

## Slide 4
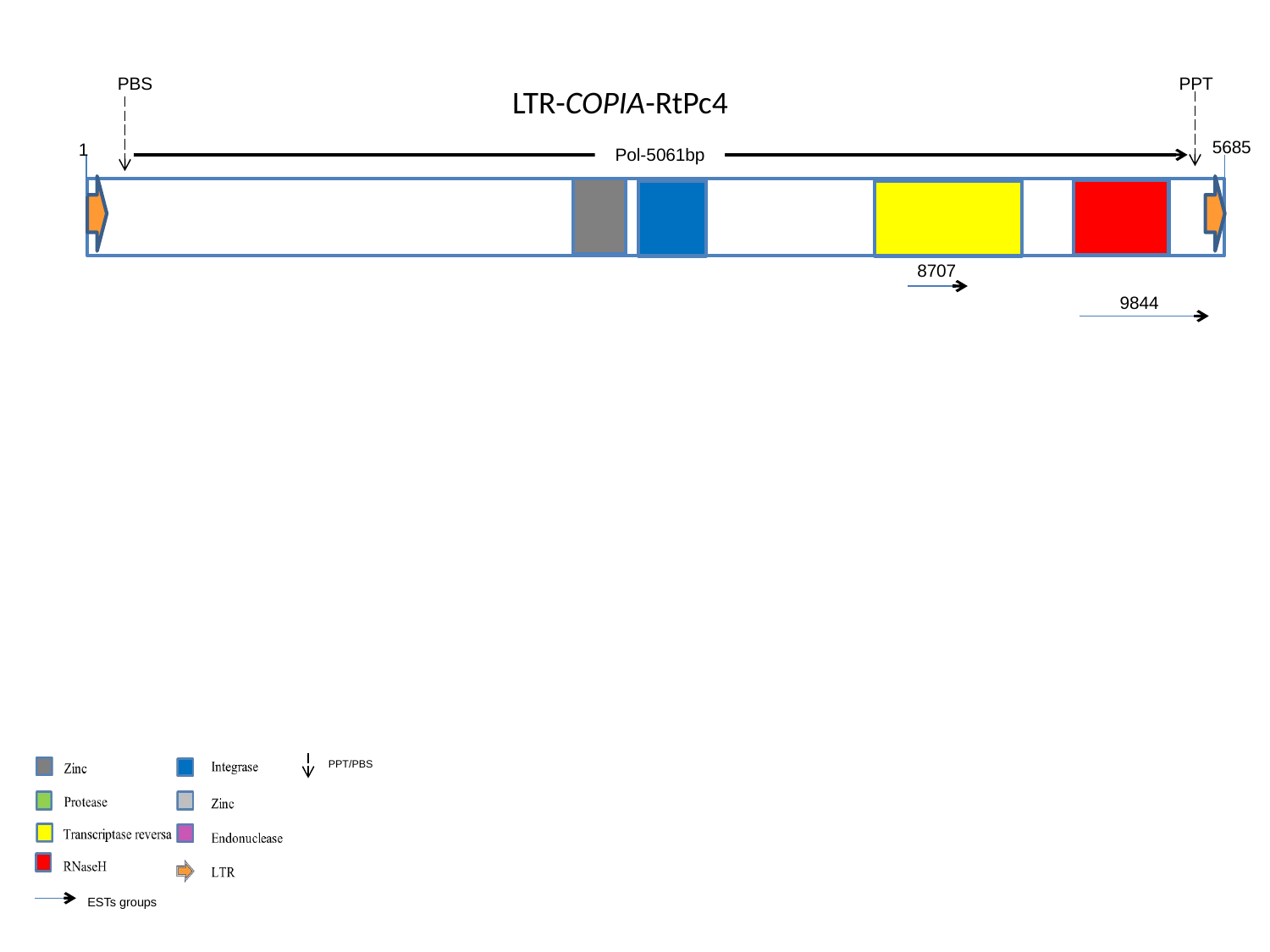

PBS
PPT
LTR-COPIA-RtPc4
5685
1
Pol-5061bp
8707
9844
PPT/PBS
ESTs groups

## Slide 5
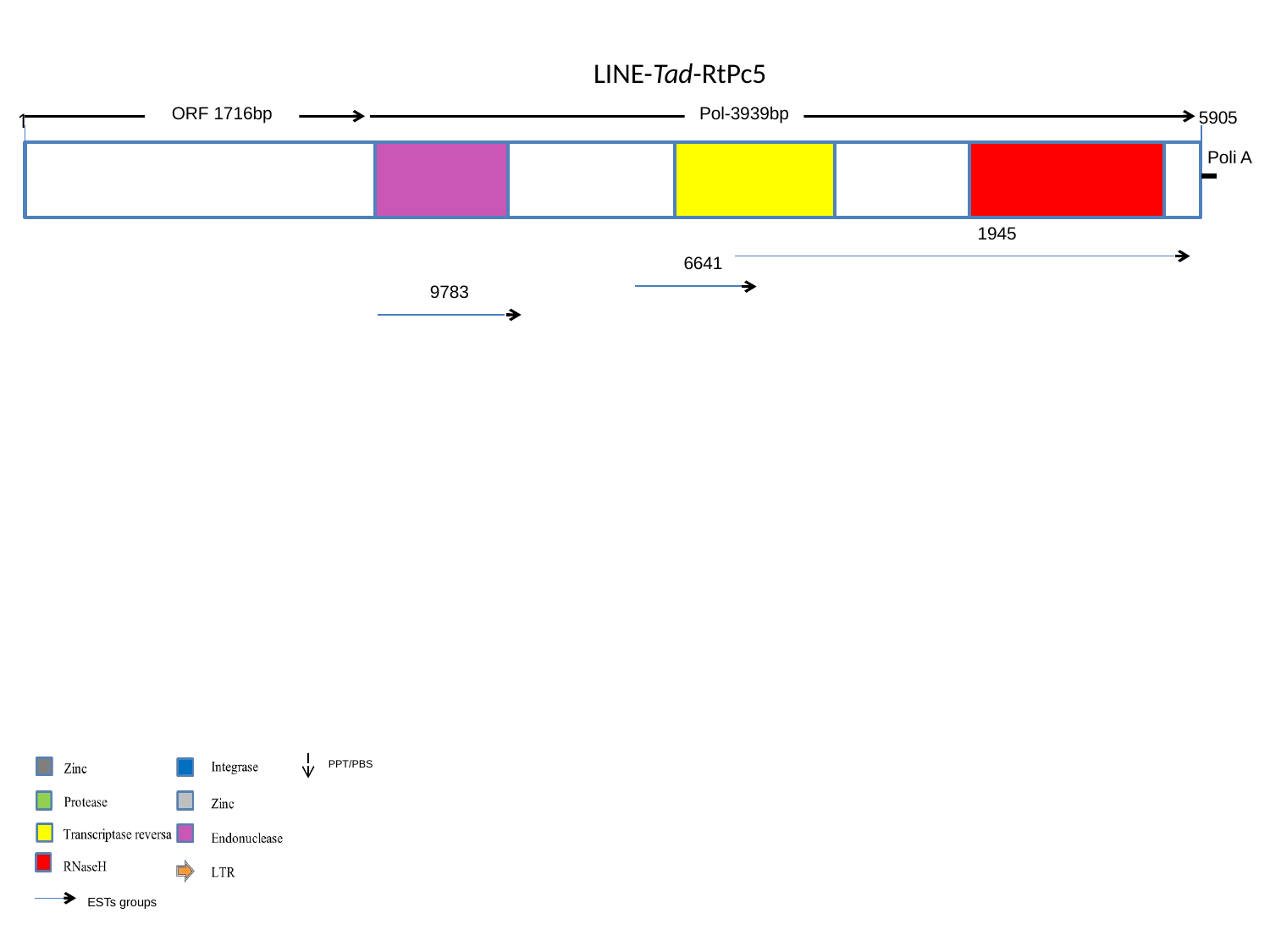

LINE-Tad-RtPc5
ORF 1716bp
Pol-3939bp
5905
1
Poli A
1945
6641
9783
PPT/PBS
ESTs groups
