# Supplementary material for: Identification and characterization of expressed retrotransposons in the genome of the Paracoccidioides species complex
Source: BMC Genomics. 2015 May 12;16(1):376. doi: 10.1186/s12864-015-1564-7 (PMC4427930; doi:10.1186/s12864-015-1564-7)
Supplement: Additional file 4: — List of all RtPc insertions in Paracoccidioides genomes. The complete list of all RtPc insertions, indicating the position of each element in the genomes of Paracoccidioides lutzii (Pb01) and Paracoccidioides brasiliensis (Pb03 and Pb18). [file 12864_2015_1564_MOESM4_ESM.doc]

|  | **Characteristics** | | | | | |  |
| --- | --- | --- | --- | --- | --- | --- | --- |
|  |  | **LTR** | |  |  |  |  |
|  | **Size (bp)** |  |  | **ORF** | **PBS** | **PPT** | **Poly-A** |
|  |  |  |  | **Gag/Pol** |  |  | **Tail** |
|  |  | **5'** | **3'** |  |  |  |  |
| RtPc1 | 5740 | 1..246 | 5495..5740 | 363..5464 | 266..276 | 5427..5444 | - |
| RtPc2 | 5412 | 1..188 | 5225..5412 | 194..4978 | 207..221 | 5186..5204 | - |
| RtPc3 | 5181 | 1..103 | 5079..5181 | 379..4965 | 164..182 | 5064..5078 | - |
| RtPc4 | 5685 | 1..98 | 5588..5685 | 403..5463 | 178..192 | 5523..5542 | - |
| RtPc5 | 5905 | - | - | 2..5681 | - | - | 5880..5905 |

Supplementary file 4 - Characteristics of RtPc elements identified in *Paracoccidioides* genomes.

| LTR – long terminal repeats |
| --- |
| ORF – open reading frame |
| PBS – primer binding site |
| PPT – poly purine tract |
