# Supplementary material for: Identification and characterization of expressed retrotransposons in the genome of the Paracoccidioides species complex
Source: BMC Genomics. 2015 May 12;16(1):376. doi: 10.1186/s12864-015-1564-7 (PMC4427930; doi:10.1186/s12864-015-1564-7)
Supplement: Additional file 6: — Primer sequences used to amplify RtPc elements by PCR and RT-PCR. Table with the sequences of the primers used to amplify RtPc elements by PCR and RT-PCR. [file 12864_2015_1564_MOESM6_ESM.docx]

**Estimation of the insertion time of LTR retrotransposons elements in *Paracoccidioides* *lutzii* and *Paracoccidioides brasiliensis* genome**

|  | D | Time since insertion  (My) |
| --- | --- | --- |
| RtPc1_ 1.16_Pb01 | 0 | 0 |
| RtPc1_ 1 19_Pb01 | 0 | 0 |
| RtPc1_ 1 22_Pb01 | 0 | 0 |
| RtPc1_1 29_Pb01 | 0,0082 | 1,21 |
| RtPc1_1.1_Pb18 | 0 | 0 |
| RtPc1_1.4_Pb18 | 0 | 0 |
| RtPc1_1.6_Pb18 | 0 | 0 |
| RtPc1_1.7_Pb18 | 0 | 0 |
| RtPc1_1.10_Pb18 | 0,0041 | 0,592 |
| RtPc1_1 11_Pb18 | 0 | 0 |
| RtPc1_ 1.14_Pb18 | 0 | 0 |
| RtPc1_ 1.3_Pb01 | 0 | 0 |
| RtPc2_1.5_Pb18 | 0,01 | 1,48 |
| RtPc3 Pb03 1.7 | 0,019 | 2,81 |
| RtPc3 Pb03 1.17 | 0,0192 | 2,84 |
| RtPc3 Pb03 1.1 | 0 | 0 |
| RtPc3 Pb18 1.5 | 0 | 0 |
| RtPc4 Pb18 1.13 | 0 | 0 |

My – million years

Time since insertion (MY) 🡪T=D/2t

Were:

T = the estimated time elapsed since the insertion

D= the estimated LTR divergence

t=0.006 substitution rate per site per Million year
